# Re‑fracture risk following implant removal in consolidated hip fractures: a multicenter retrospective cohort study of 575 patients

**DOI:** 10.1007/s00068-026-03258-6

**Published:** 2026-07-06

**Authors:** Jabbar Mohammed, Viktor Mili-Schmidt, Mats Wadsten, Hans Juto, Olof Wolf, Sead Crnalic, Olof Sköldenberg, Sebastian Mukka, Per Fischer

**Affiliations:** 1 Department of Diagnostics and Intervention (Orthopaedics), Umeå University, Umeå, Sweden; 2 Department of Clinical Sciences at Danderyd Hospital, Division of Orthopaedics, Karolinska Institute, Stockholm, Sweden; 3 Department of Surgical Sciences, Section of Orthopedics, Uppsala University, Uppsala, Sweden; 4 Faculty of Medicine and Health, Örebro University, Örebro, Sweden

**Keywords:** Hip fracture, Implant removal, Re-fracture risk, Internal fixation, Hip arthroplasty, Hip complication, Lateral hip pain

## Abstract

**Purpose:**

The risk of re-fracture after implant removal in healed hip fractures remains a clinical concern. This study aimed to determine the incidence of re-fracture following implant removal after osteosynthesis of a hip fracture.

**Methods:**

We conducted a retrospective multicenter cohort study including patients aged ≥ 50 years who underwent implant removal between 2003 and 2023 after radiologically confirmed consolidation of a hip fracture. Patients were identified using procedural codes. Baseline variables included age, sex, ASA classification, fracture type (femoral neck or trochanteric), and implant type (pins/screws, sliding hip device [SHD], or short/long cephalomedullary nail [CMN]). Patients were followed from implant removal until re-fracture, conversion to arthroplasty, death, or end of follow-up, with a minimum follow-up of 1 year. Cox proportional hazards regression was used to assess associations between implant type and re-fracture risk, adjusting for age and sex. Because the proportional hazards assumption was violated, a time-stratified Cox regression and restricted mean survival time analyses were applied.

**Results:**

A total of 575 patients (median age 73 years, IQR 65–81) were included, with a median follow-up of 53 months (IQR 18–100). Lateral hip pain was the most common indication for implant removal (72.5%). The overall re-fracture incidence was 10.4%. Risk varied by implant type: 7.1% after pins/screws, 10.5% after SHD, 18.3% after short CMN, and 15.8% after long CMN removal. Median time to re-fracture was 1.5 months, and 52% occurred after minimal or no trauma. Most re-fractures (85%) occurred within 90 months. Removal of CMNs was associated with a higher re-fracture risk compared with pins/screws (HR 2.79; 95% CI 1.52–5.13), whereas SHD removal was not.

**Conclusion:**

Implant removal after consolidated hip fractures carries a measurable risk of re-fracture, most pronounced after CMN removal. These findings highlight the importance of careful patient selection and could aid in decision-making when considering implant exchange or removal following osteosynthesis.

**Levels of evidence:**

IV, retrospective observational cohort study.

## Introduction

Hip fractures represent a major health burden in older adults and are associated with substantial morbidity and mortality [1, 2]. Consequently, as a substantial proportion of hip fractures are treated with internal fixation, considerable research has focused on complications related to fixation devices and surgical techniques used in hip fracture treatment [3, 4]. In some cases, implant removal, re-osteosynthesis, or conversion to hip arthroplasty is required due to indications such as infection, mechanical failure, non-union, or implant breakage [5, 6]. However, even after fracture healing, implants are occasionally removed for relative indications, including persistent lateral hip pain, mobility limitations, or patient and surgeon preference [7, 8]. Despite being a relatively common clinical practice, implant removal after consolidated hip fractures remains poorly studied. Biomechanical studies have suggested that removal of sliding hip devices (SHDs) and cephalomedullary nails (CMNs) could reduce femoral stability and thereby increase the risk of re-fracture [9, 10]. Nevertheless, the available clinical evidence remains limited and inconsistent. Reported re-fracture rates vary considerably between studies, ranging from 8.1% to 14% [6, 11, 12], while other studies have found no significant association between implant removal and subsequent fracture risk [13]. This uncertainty is particularly concerning in older patients with osteoporosis, impaired bone quality, and a previous fracture, who may be especially vulnerable to secondary fractures after implant removal [11, 14–16]. Furthermore, standardized guidelines regarding implant removal after healed hip fractures are lacking. Therefore, this study aimed to determine the incidence of re-fracture following implant removal after osteosynthesis of hip fractures.

## Patients and methods

### Study design and setting

This retrospective cohort study included patients who underwent implant removal between January 2003 and December 2023 after osteosynthesis of a hip fracture at six Swedish hospitals (Uppsala, Danderyd, Karlstad, Örebro, Sundsvall, and Sunderby). This design was chosen to enable the inclusion of a larger patient cohort with sufficient follow-up time to evaluate the relatively uncommon outcome of re-fracture following implant removal. Data were collected through review of medical records and radiographic assessments. Reporting adhered to the STROBE (Strengthening the Reporting of Observational Studies in Epidemiology) guidelines [17].

### Patients and data collection

**Fig. 1:**
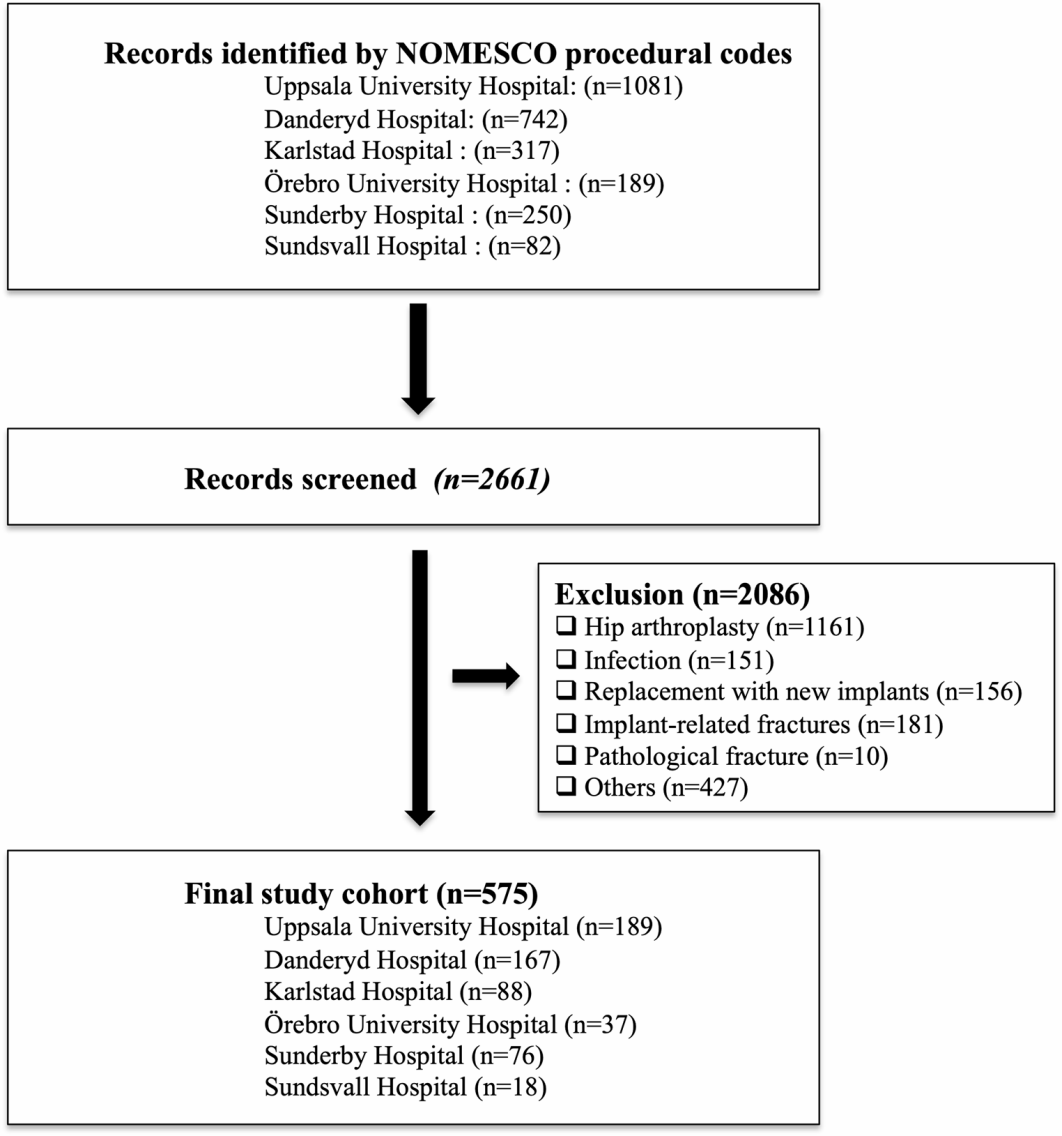
Flowchart illustrating patient selection: 2,661 records were reviewed, resulting in a final study cohort of 575 patients from six Swedish hospitals

Patients aged ≥ 50 years, representing the age group commonly used in osteoporosis and fragility fracture research [16, 18], who underwent implant removal after radiologically confirmed consolidation of a hip fracture were identified using NOMESCO (Nordic Medico-Statistical Committee) procedural codes for femoral implant removal (NFU39/49/89/99) (Fig. 1). Baseline variables included age, sex, ASA classification, fracture type, and implant type (hook pins/screws, SHD, short or long CMNs). Clinical details of implant removal were extracted, including date, indication (e.g., lateral hip pain, groin pain, mobility issues), time from index surgery to removal, and whether removal was partial (femoral neck fixation) or complete. Re-fracture data included occurrence (yes/no), date, type, and location, classified using Garden (femoral neck) and AO/OTA (trochanteric/shaft) systems [19, 20]. Garden classification was dichotomized into undisplaced (Garden I–II) and displaced fractures (Garden III–IV). ASA classification was grouped into ASA I–II and ASA III–IV to differentiate between patients with absent or mild systemic disease and those with substantial systemic comorbidity. Subsequent treatments, reoperations, complications (e.g., infection, wound healing), and mortality were recorded. Radiographs confirmed fracture healing and implant type. Patients were followed until conversion to arthroplasty, re-fracture, death, or end of follow-up, whichever occurred first. Exclusion criteria were bilateral fractures, contralateral fractures after the index event, pathological or stress fractures, previous peri-implant fractures, infection-related removals, arthroplasty at removal, and incomplete records.

### Definition of re-fracture

A re-fracture was defined as a femoral fracture occurring after implant removal from a previously consolidated hip fracture and located in proximity to the prior implant. Re-fractures were classified anatomically as femoral neck, trochanteric, or femoral shaft fractures. The date of re-fracture and details of subsequent surgical interventions were systematically recorded.

### Implants and surgery

Hip fractures were treated with internal fixation methods according to fracture type and surgeon preference. Trochanteric fractures were commonly managed with SHDs (DHS, (DePuy Synthes^®^); Hansson Twin Hook (Swemac Orthopaedics AB, Linköping, Sweden), or CMNs (Gamma3^®^, Stryker, Kalamazoo, MI, USA; PFN/PFN-A, DePuy Synthes^®^, Trigen Intertan, Smith & Nephew, Memphis, TN, USA). Femoral neck fractures (FNFs) were treated with the Hansson Hook Pin System and Hansson Pinloc (Swemac Orthopaedics AB, Linköping, Sweden), or Olmed screws (Zimmer Biomet, Warsaw, IN, USA). In selected cases, plate fixation was supplemented with an antirotation screw or pin. After radiographic confirmation of fracture healing, implants were removed for clinical indications. Removal procedures varied by implant type, with device-specific instruments used. All patients received prophylactic antibiotics per institutional protocol and were mobilized with full weight-bearing postoperatively. Procedures were performed by consultant orthopedic surgeons or supervised residents.

### Outcome

The primary outcome was the incidence of re-fracture following implant removal after radiologically consolidated hip fracture. Secondary outcomes included potential risk factors for re-fracture (age, sex, fracture type, implant type). Additional key variables were the indication for implant removal, time from primary surgery to removal, time from removal to re-fracture, and surgical management of re-fractures.

### Statistical analysis

Normality of continuous variables was assessed using the Shapiro–Wilk test. Normally distributed data are presented as mean (SD), and non-normally distributed data as median (interquartile range, IQR). Categorical variables are reported as frequencies and percentages. Group differences were analyzed using the Mann-Whitney U test for continuous variables and Pearson’s chi-square test for categorical variables. Kaplan-Meier survival curves illustrated time to re-fracture across implant groups (hook pins/screws, SHD, CMNs). Because curves for SHD and hook pins/screws intersected at ~ 100 months, proportional hazards assumptions were considered violated. Cox regression analyses were therefore stratified by re-fracture status: within 90 months and after 90 months, with hazard ratios (HRs) and 95% confidence intervals (CIs) adjusted for age and sex. Restricted mean survival time (RMST) up to 90 months was also calculated to quantify absolute differences in re-fracture-free survival. Statistical significance was set at *p* < 0.05 (two-tailed). Analyses were performed using SPSS version 29 (Mac) and R version 4.3.2 (R Foundation for Statistical Computing).

## Results

### Patients and descriptive data

Of the 2,661 implant removals initially screened, 575 patients remained for the final analysis after application of the inclusion and exclusion criteria (Fig. 1). The median age was 73 years (IQR, 65–81), and 65% were female. Median follow-up was 53 months (IQR 18–100). Most removals were performed at Uppsala (189 cases, 33%) and Danderyd (167 cases, 29%). Initial fracture types included FNFs (Garden I-IV) in 63% (360 cases) and trochanteric fractures (AO/OTA 31-A1-A3) in 37% (213 cases) (Table 1).

**Table 1:** Baseline characteristics of the total cohort (*n* = 575). Continuous variables are expressed as median (IQR), and categorical variables as counts and percentages

Variable	Total Cohort (*n* = 575)
**Sex, ** ***n*** ** (%)**	
Female	378 (65.7)
Male	197 (34.3)
**Age**,** median (IQR)**	73 (65–81)
**ASA Category**,** n (%)**	
1–2	327 (56.9)
3–5	247 (43.0)
Missing	1 (0.2)
**Fracture side**,** n (%)**	
Left	294 (51.1)
Right	281 (48.9)
**Hospital**,** n (%)**	
Uppsala	189 (32.9)
Danderyd	167 (29.0)
Örebro	37 (6.4)
Sundsvall	18 (3.1)
Sunderby	76 (13.2)
Karlstad	88 (15.3)
**Type of initial fracture n (%)**	
FNF, Garden I–II	271 (47.1)
FNF, Garden III–IV	89 (15.5)
TF AO/OTA 31-A1	61 (10.6)
TF AO/OTA 31-A2	88 (15.3)
TF AO/OTA 31-A3	64 (11.1)
Missing	2 (0.3)

FNF = femoral neck fracture; TF = trochanteric fracture; ASA = American Society of Anesthesiologists

### Implant removal details

Median time from initial fracture fixation to implant removal was 17 months (IQR 12–28). Most patients (58.8%, 338/575) underwent removal of hook pins or screws, while 26.3% (151/575) had CMNs removed and 14.8% (85/575) had SHDs removed (Table 2). Lateral hip pain was the predominant indication for implant removal (72.5%), followed by lateralization of fixation hardware (17.7%). Less frequent indications included medialization (4.3%), pain due to avascular necrosis (AVN, 2.4%), surgeon preference (1.2%), and patient request (1.6%). Complete implant removal was performed in 80.2% (461/575) of patients, whereas partial removal accounted for 19.8% (114/575), most commonly involving CMNs.

**Table 2:** Indications for implant removal and type of removal (partial vs. total)

Variable	Total (*n* = 575)	No re-fracture (*n* = 515)	Re-fracture (*n* = 60)
**Indication for implant removal, ** ***n*** ** (%)**			
Lateral hip pain	417 (72.5)	375 (72.8)	42 (10.1)
Patient request	9 (1.6)	7 (1.4)	2 (22.2)
Surgeon preference	8 (1.4)	8 (1.6)	0 (0)
Lateralization of femoral fixation hardware	101 (17.6)	90 (17.5)	11 (10.9)
Medialization of femoral fixation hardware	25 (4.3)	22 (4.3)	3 (12.0)
Pain secondary to AVN	15 (2.6)	13 (2.5)	2 (13.3)
**Type of removal**,** n (%)**			
*Total removal*	461 (80.2)	418 (81.2)	43 (9.3)
*Partial removal*	114 (19.8)	97 (18.8)	17(14.9)
LIH/Olmed (1 of 2 screws)	5 (0.9)	5 (1.0)	0 (0)
DHS/TH (screw or TH blade)	6 (1.0)	6 (1.2)	0 (0)
CMN (removal of blade/screw)	70 (12.2)	54 (10.5)	16 (22.9)
CMN (1 of 2 screws, Trigen, PFN)	18 (3.1)	18 (3.5)	0 (0)
DHS + (hook pin/screw)	12 (2.1)	12 (2.3)	0 (0)
CMN (both blade/screw)	3 (0.5)	2 (0.4)	1 (33.3)
**Type of Implant**,** n (%)**			
***Hook pins/screws***			
LIH	78 (13.6)	72 (14.0)	6 (7.7)
Olmed	251 (43.7)	234 (45.4)	17 (6.8)
Pinlock	9 (1.6)	8 (1.6)	1 (11.1)
***Sliding hip device***			
Twin hook	23 (4.0)	21 (4.1)	2 (8.7)
DHS	37 (6.4)	31 (6.0)	6 (16.2)
DHS plus additional screw	20 (3.5)	19 (3.7)	1 (5.0)
Twin hook plus additional hook pin or screw	6 (1.0)	6 (1.2)	0 (0)
***Cephalomedullary nails***			
Short TFNA	2 (0.3)	2 (0.4)	0 (0)
Short Gamma nail	55 (9.6)	44 (8.5)	11 (20.0)
Short Trigen & PFN nails	7 (1.2)	4 (0.8)	3 (42.9)
Short PFN	27 (4.7)	22 (4.3)	5 (18.5)
Short PFNA	22 (3.8)	20 (3.9)	2 (9.1)
Long PFNA	1 (0.2)	1 (0.2)	0 (0)
Long Gamma nail	34 (5.9)	28 (5.4)	6 (17.6)
Long PFN	1 (0.2)	1 (0.2)	0 (0)
Long Trigen and PFN nails	2 (0.3)	2 (0.4)	0 (0)

AVN = Avascular Necrosis; LIH = Lars Ingvar Hansson Pin(hook); DHS = Dynamic Hip Screw; PFN = Proximal Femoral Nail; PFNA = Proximal Femur Nail Antirotation; TFNA = TFN-ADVANCED Proximal Femoral Nailing System

### Re-fracture

Re-fractures occurred in 60 patients (10.4%, 60/575). Rates were 7.1% (24/338) after removal of pins/screws, 18.3% (21/113) after short CMN removal, 15.8% (6/38) after long CMN removal, and 10.5% (9/86) following SHD removal. Median time to re-fracture was 1.5 months (IQR 0–43), with 52% occurring after minimal or no trauma. Minimal or no trauma was defined as either a low energy event without a documented fall, such as a simple slip, or the absence of a clearly documented precipitating trauma mechanism, for example increased hip pain occurring during walking. Patients with re-fracture had implants removed significantly earlier after primary surgery than those without (median 13 vs. 17 months, *p* = 0.018). Re-fractures were more common in AO 31-A2 and A3 fracture patterns compared with FNFs. Most occurred after complete implant removal. Among partial removals, all 17 re-fractures followed removal of femoral neck fixation in CMN constructs, yielding a 23.2% re-fracture rate in this subgroup (Table 2). Implant-specific subgroup analyses were limited by small numbers; numerically higher re-fracture rates were observed after partial removal of short CMN constructs (Table 2). Of the 60 re-fractures, 63.3% (38/60) were FNFs, 30.0% (18/60) trochanteric, and 6.7% (4/60) femoral shaft fractures (Table 3). None of the recorded re-fractures occurred intraoperatively.

**Table 3:** Patients with re-fractures stratified by anatomical location and type of implant removed

Implant type	FNF, Garden I–II	FNF, Garden III–IV	TF-A1	TF-A2	TF-A3	Femoral shaft	Total (*n*)
Hook pins or Screws	4	8	5	5	1	0	**23**
Short CMNs	3	15	1	1	0	1	**21**
SHD	1	1	1	2	2	1	**8**
Long CMNs	2	3	0	0	0	1	**6**
SHD plus additional screw	0	0	0	0	0	1	**1**
Other (Pinloc)	0	1	0	0	0	0	**1**
Total (n)	10	28	7	8	3	4	**60**

FNF = Femoral Neck Fracture; TF = Trochanteric Fracture; SHD = Sliding Hip Device; CMN = Cephalomedullary Nail

### Survival analysis

**Fig. 2:**
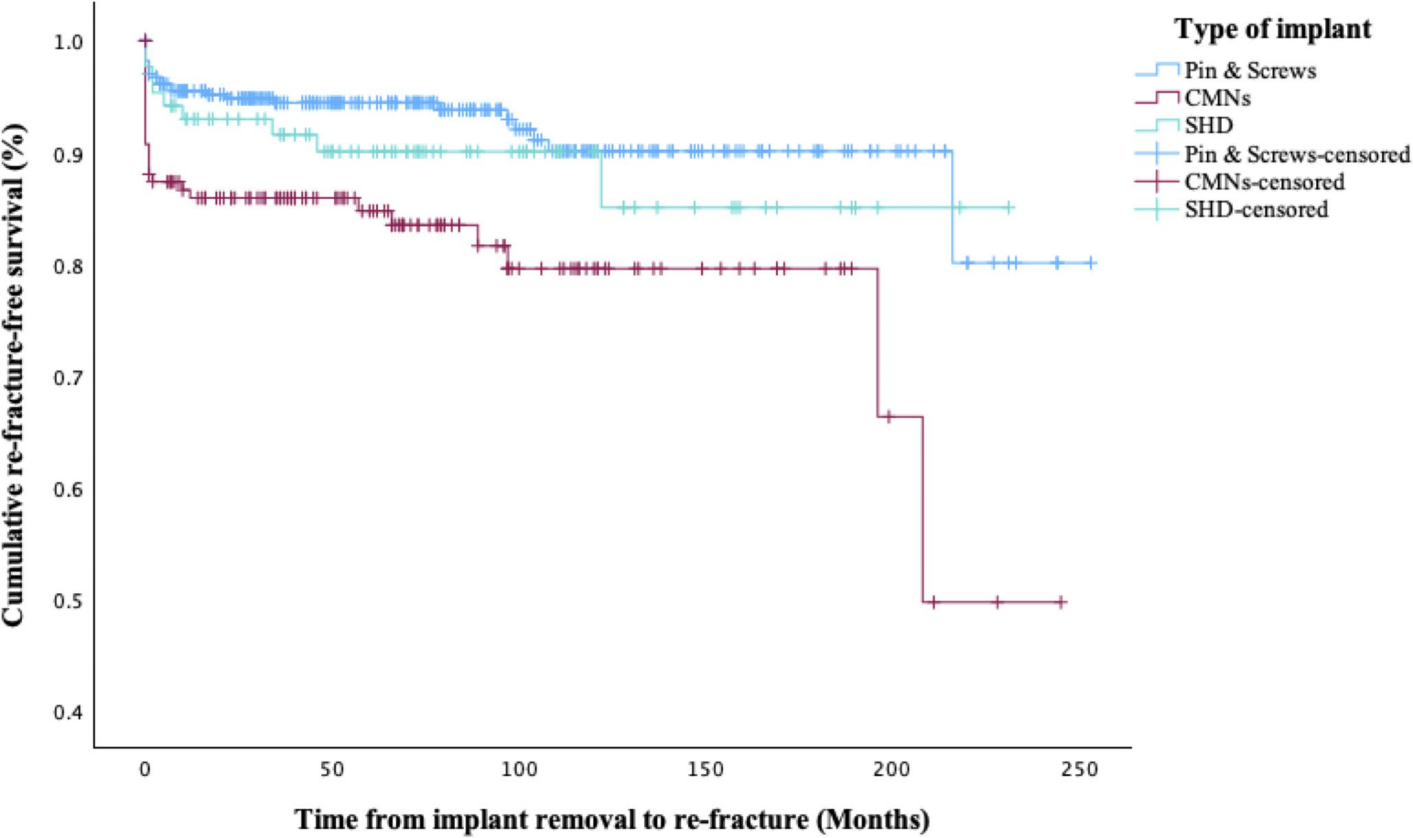
Kaplan-Meier curves illustrating cumulative incidence of re-fracture after implant removal, stratified by implant type. The curves intersect at approximately 100 months, indicating a time-dependent change in hazard ratio. Differences between groups were assessed using the log-rank test (p = 0.01)

Kaplan–Meier curves diverged early but crossed at ~ 100 months for pins/screws and SHDs (Fig. 2). Because of non-proportional hazards, the Cox regression analyses were stratified into 0–90 months and > 90 months (Fig. 2). Within the first 90 months, 51 re-fractures occurred. In adjusted models (age, sex, implant type), CMN removal was associated with a significantly higher risk of re-fracture compared with pins/screws (adjusted HR [aHR] = 2.79; 95% CI, 1.52–5.12). SHD removal did not statistically significantly increase the risk of re-fracture compared with pins/screws (aHR = 1.91; 95% CI 0.82–4.44). Beyond 90 months, only nine re-fractures were observed, with no significant associations between implant type and re-fracture risk (*p* > 0.05) (Tables 4 and 5).

**Table 4:** Cox regression analysis of predictors of re-fracture within 90 months after implant removal

Variable	Total* (n)*	Re-fracture *(n)*	Crude HR (95% CI)	*P*-value	Adjusted HR (95% CI)	*P*-value
**Age**,** median (IQR) years**	75 (66–83)	51	1.01 (0.99–1.03)	0.30	1.00 (0.98–1.03)	0.65
**Sex**						
Female	269	41	1.00 (ref)		1.00 (ref)	
Male	137	10	2.08 (1.04–4.16)	0.03	1.44 (1.01–2.05)	0.04
**Implant removed**				0.002		0.004
Hook pins or screws	239	19	1.00 (ref)		1.00 (ref)	
Twin Hook / DHS	56	8	1.74 (0.76–3.98)	0.18	1.91 (0.82–4.44)	0.13
CMNs	111	24	2.83 (1.55–5.19)	< 0.001	2.79 (1.52–5.13)	< 0.001

Hazard ratios (HRs) are presented with 95% confidence intervals (CIs). Variables with *p* < 0.05 in the adjusted model were considered statistically significant

**Table 5:** Cox regression analysis of predictors of re-fracture after 90 months following implant removal

Variable	Total* (n)*	Re-fracture (*n*)	Crude HR (95% CI)	*P*-value	Adjusted HR (95% CI)	*P*-value
**Age**,** median (IQR) years**	69 (62–78)	9	1.03 (0.96–1.11)	0.35	1.03 (0.96–1.11)	0.37
**Sex**						
Female	109	5	1.00 (ref)		1.00 (ref)	
Male	60	4	0.67 (0.34–1.31)	0.25	0.59 (0.29–1.19)	0.14
**Implant removed**				0.75		0.61
Hook pins or screws	99	5	1.00 (ref)		1.00 (ref)	
Twin Hook / DHS	30	1	0.66 (0.77–5.68)	0.70	0.59 (0.63–5.60)	0.65
CMNs	40	3	1.48 (0.35–6.23)	0.58	1.76 (0.40–7.72)	0.45

Hazard ratios (HRs) are presented with 95% confidence intervals (CIs), and variables with *p* < 0.05 in the adjusted model were considered statistically significant

RMST up to 90 months was lowest for CMN removal. The RMST difference between CMN and pins/screws was − 12.0 months (95% CI − 18.9 to − 5.1), while the difference between SHD and pins/screws was − 2.24 months (95% CI − 9.9 to 5.4). These results were consistent with the Cox regression findings within the first 90 months after implant removal.

### Treatment of re-fracture

Femoral neck re-fractures (*n* = 38) were primarily treated with hip arthroplasty. Of these, 52% (20/38) underwent total hip arthroplasty and 13.2% (5/38) hemiarthroplasty. One patient was treated with screw fixation, one with a CMN, and one with femoral neck fixation through the existing implant. Trochanteric re-fractures (*n* = 18) were most often managed with internal fixation, including SHD using the TwinHook construct (29%, 5/18), short CMN (29%, 5/18), long CMN (29%, 5/18). One patient received screw fixation, and one underwent total hip arthroplasty. Femoral shaft fractures (*n* = 4) were treated with plate fixation (50%, 2/4) or CMN (50%, 2/4). Overall, non-operative management was used in 13% (8/60) of cases, primarily in frail patients, and the Girdlestone procedure was performed in 5% (3/60).

## Discussion

In this large multicenter cohort of 575 patients, implant removal after consolidated hip fractures was associated with a 10.4% re‑fracture rate, with the highest risk following CMN removal. Although re-fracture after implant removal has been reported previously, the magnitude and early occurrence of re-fracture observed in this population-based cohort were unexpected. Prior literature has largely been limited to small case series, whereas our findings demonstrate that re-fracture is a clinically relevant and non-negligible complication, particularly within the first months after implant removal. Re‑fractures occurred mainly in the femoral neck, followed by trochanteric and subtrochanteric regions, and were concentrated in the early postoperative period. These findings confirm that implant removal is not a benign procedure and highlight the need for careful patient selection. To our knowledge, this is the largest study to date examining re‑fracture risk after implant removal in consolidated hip fractures.

**Fig. 3:**
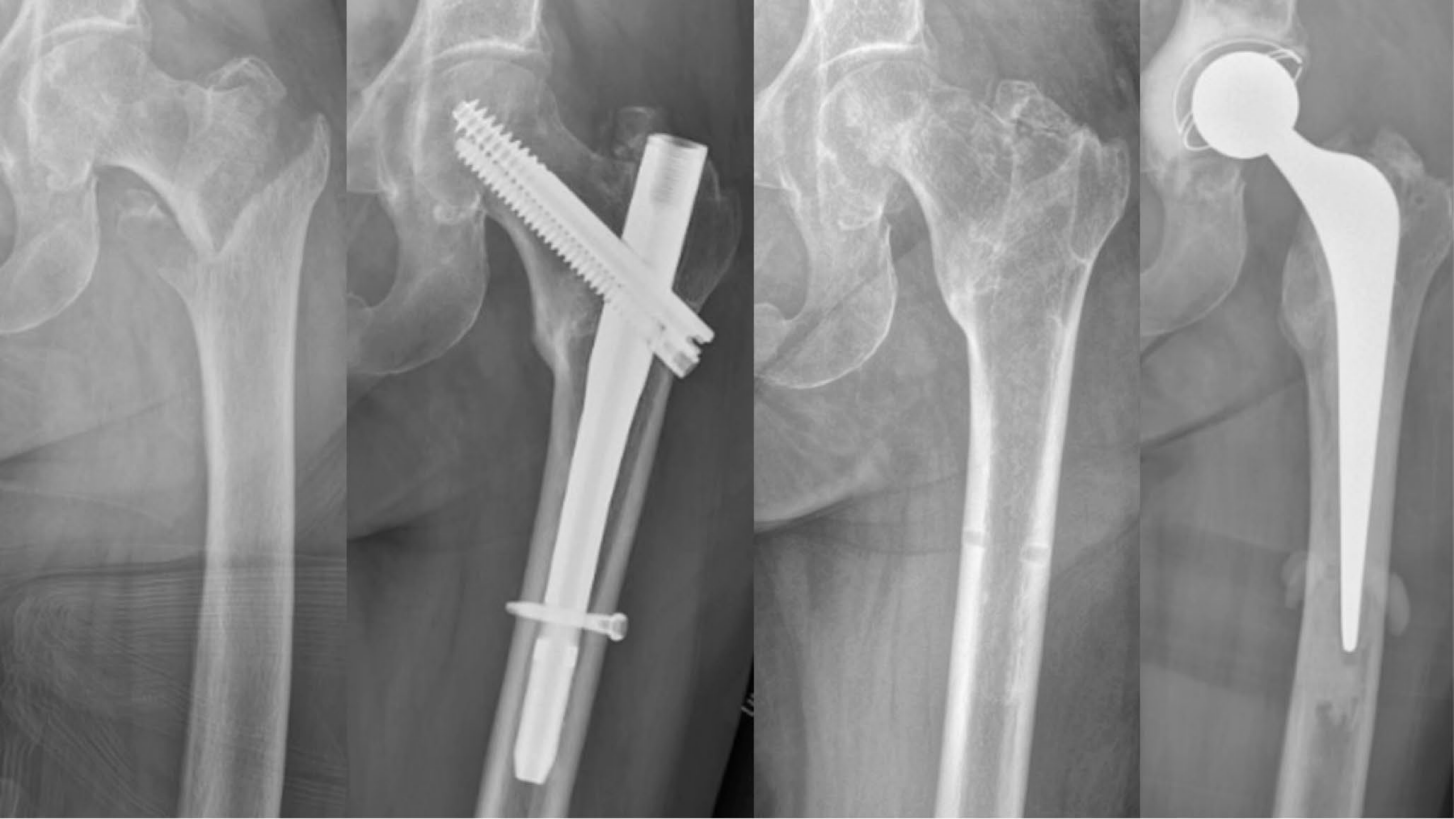
Trochanteric hip fracture treated with a short Trigen nail. The implant was removed 25 months after fixation due to lateral hip pain. Within one week postoperatively, the patient developed groin pain and an inability to bear weight. Radiographs obtained seven days after extraction revealed a femoral neck fracture, subsequently managed with total hip arthroplasty

Our results align with earlier reports indicating that removal of load-sharing implants significantly weakens the proximal femur [8, 11, 12, 14, 15, 21]. Previous reports have largely focused on femoral neck re-fractures after removal of implants used for trochanteric fractures. A systematic review of 114 implant removals reported a median femoral neck re-fracture rate of 14.5%, with 87% occurring atraumatically within three weeks [12]. Driessen and Goossens described five atraumatic re-fractures within three weeks of CMN removal in women with osteoporosis [15], while Yoon et al. documented a 9% femoral neck re-fracture rate within one month following SHD removal [11]. In our cohort, femoral neck re-fractures occurred in 3.8% (13/338) following hook pin/screw removal, 2.3% (2/86) after SHD removal, and 15.2% (23/151) after CMN removal (Fig. 3). Given the median age and sex distribution of the cohort, and that all patients had sustained a hip fracture, a substantial proportion of the study population had osteopenia or osteoporosis. This assumption is supported by Astrand et al., who found that only 13% of patients over 50 years of age presenting with fragility fractures had normal bone mineral density [16]. Re-fractures and subsequent fractures are expected in a group such as this, and we have now assessed re-fracture risk after implant removal in patients with previous hip fractures.

Re-fractures distal to the femoral neck after implant removal have rarely been reported, with evidence largely limited to isolated case reports [22]. In our cohort, 18 trochanteric re-fractures were identified: 11 after removal of hook pins/screws, 5 after DHS removal, and 2 after CMN removal. We also observed 4 femoral shaft re-fractures, two following SHD removal and 2 after CMN removal. Only 4 of these 22 distal re-fractures occurred atraumatically or after minimal trauma. Published cases include a report by Jin et al., who described an atraumatic trochanteric re-fracture after CMN removal in a patient with radiographically confirmed healing [22].

Biomechanical studies provide a plausible explanation for the re-fracture patterns observed after implant removal in consolidated trochanteric fractures. Schwarz et al. demonstrated substantially reduced failure loads and altered stress distribution in the femoral neck after CMN removal compared with intact femur [9]. Kukla et al. similarly reported significantly lower failure loads after CMN removal than after SHD removal, with most fractures involving the femoral neck [6]. These experimental findings are consistent with our clinical results, in which re-fractures predominantly involved the femoral neck and rates were higher after CMN removal than after SHD removal. Notably, re-fracture rates were higher after removal of only the femoral-neck fixation components of CMNs (23.2%) compared with complete implant removal (16.4%). A possible explanation is that partial removal leaves the medullary nail in situ while creating a residual femoral neck defect after removal of the cephalic fixation, potentially resulting in altered load transfer and stress concentration within an already weakened femoral neck. In this older and potentially frail patient population, this biomechanical imbalance may further increase risk of re-fractures. However, these findings should be interpreted with caution given the limited subgroup size and retrospective design.

The indication for implant removal remains a clinical dilemma. In our cohort, 73% of removals were performed for lateral hip pain, and implant removal was generally considered in patients with persistent symptoms despite non-operative management; however, the type, duration, and response to non-operative treatment was not consistently documented and could therefore not be analyzed, nor could postoperative symptom relief following implant removal be assessed. Previous studies have reported variable outcomes: Minkowitz et al. noted symptomatic improvement in 76% of patients [23], whereas others found limited satisfaction when no alternative pain source was identified [24, 25]. Given the increased risk of re-fracture observed in our study, routine implant removal should be avoided [26]. In cases where lateralization of the femoral neck fixation hardware causes symptoms, implant exchange may represent a safer alternative, and complete healing should be confirmed with computed tomography [6, 11, 15].

Peri-implant femur fractures (PIFF) may occur in patients with retained implants. A previous study reported cumulative incidence of 0.8% (3/382) after CMN, 2.7% (25/933) after SHD, and 2.0% (13/650) after pin fixation, with a mean time interval of 27 months between primary osteosynthesis and PIFF [26]. In contrast, our study demonstrates that implant removal is associated with a higher risk of re-fracture, particularly after CMN removal, and that these re-fractures tend to occur earlier in the postoperative period. These findings underscore the importance of careful patient selection and a restrictive approach to hardware removal in patients with hip fractures.

This study has several limitations, including its retrospective design with inherent risk of bias and its smaller sample size relative to registry-based studies. In addition, healing of the initial fracture was assessed using radiographs, which may overestimate union and lead to misclassification; CT would have been preferable, but we used the available data as this was a retrospective study. Furthermore, due to the retrospective study design, detailed and standardized information regarding osteoporosis, bone mineral density, smoking, corticosteroid treatment, metabolic bone disease, and other risk factors potentially affecting bone healing and re-fracture risk was not consistently available for all patients. In addition, implant designs and treatment strategies may have evolved and been refined over the study period and differed between participating centers, potentially influencing the observed outcome. Nonetheless, important strengths offset these limitations. Sweden’s unique personal identification number enabled precise linkage across hospital databases, ensuring high data completeness. Based on the available evidence, this represents the largest cohort to date evaluating re-fracture risk after hardware removal in consolidated hip fractures, providing new insights into timing, implant-specific risk differences, and early failure patterns. Because hip fracture patients are typically older (> 65 years) and rarely relocate, the likelihood of missing re-fractures treated at other hospitals is limited.

## Conclusion

Implant removal after consolidated hip fractures carries a measurable risk of re-fracture, most pronounced after CMN removal. These findings highlight the importance of careful patient selection and could aid in decision-making when considering implant exchange or removal following osteosynthesis.

## Data Availability

Data underlying this study cannot be shared publicly due to legal restrictions under the Swedish Public Access and Secrecy Act (chapter 21, §7; chapter 25, §1). Researchers interested in accessing the dataset may contact the corresponding author to discuss data sharing in accordance with Swedish law.
